# Microplastic pollution in seawater and marine organisms across the Tropical Eastern Pacific and Galápagos

**DOI:** 10.1038/s41598-021-85939-3

**Published:** 2021-03-19

**Authors:** Alonzo Alfaro-Núñez, Diana Astorga, Lenin Cáceres-Farías, Lisandra Bastidas, Cynthia Soto Villegas, Kewrin Macay, Jan H. Christensen

**Affiliations:** 1 Virus Research & Development Laboratory, Statens Serum Institut, Artillerivej 5, 2300 København S, Denmark; 2 Section for Evolutionary Genomics, GLOBE Institute, University of Copenhagen, Øster Farimagsgade 5, 1353 Copenhagen K, Denmark; 3 Escuela de Ciencias Biológicas, Pontificia Universidad Católica del Ecuador, Av. 12 de Octubre 1076, 17-01-2184 Apartado, Quito Ecuador; 4 Grupo de Investigación en Sanidad Acuícola, Inocuidad y Salud Ambiental, Escuela de Acuicultura y Pesquería, Facultad de Ciencias Veterinarias, Universidad Técnica de Manabí, Ciudadela Universitaria, Leonidas Plaza EC131402, Bahía de Caráquez, Ecuador; 5 AquaCEAL Corp. Urb, Las Palmeras, av. Capitán Byron Palacios y General Quisquis, #8 EC230101, Santo Domingo de los Colorados, Ecuador; 6 School of Biological Science and Engineering, Yachay Tech University, Urcuquí, Imbabura Ecuador; 7 Department of Plant and Environmental Sciences, University of Copenhagen, Thorvaldsensvej 40, 1871 Frederiksberg, Denmark

**Keywords:** Ecology, Environmental sciences, Natural hazards, Ocean sciences, Endocrinology, Risk factors, Signs and symptoms, Nanotoxicology, Techniques and instrumentation

## Abstract

Detection of plastic debris degrading into micro particles across all oceanic environments and inside of marine organisms is no longer surprising news. Microplastic contamination now appears as one of the world’s environmental main concerns. To determine the levels of microplastic pollution at sea, water samples were collected across a 4000 km-trajectory in the Tropical Eastern Pacific and the Galápagos archipelago, covering an area of 453,000 square kilometres. Furthermore, 240 specimens of 16 different species of fish, squid, and shrimp, all of human consumption, were collected along the continental coast. Microplastic particles were found in 100% of the water samples and marine organisms. Microplastic particles ranging from 150 to 500 µm in size were the most predominant. This is one of the first reports simultaneously detecting and quantifying microplastic particles abundance and their impact on marine organisms of this region.

## Introduction

Plastics of all sizes have become the most dominant form of marine litter and it has been estimated that at least 5.25 trillion plastic particles weighing above 268,000 tons have been discarded into the Oceans^1^. Moreover, according to the 2017 United Nations Environment Assembly (UNEP) an estimate of 4.8–12.7 million metric tons of plastic are introduced to the oceans annually^2^. The low cost, lightweight, strength and durability of plastics are properties that make them suitable for manufacture on a wide range of daily use products. Virtually everything is made of plastic nowadays. However, the high demand and inappropriate disposal of plastic materials have led to their dispersion and accumulation into the environment^3^. For example, during the current COVID-19 pandemic the worldwide production and disposal of face masks as well as other plastic laboratory and medical materials have drastically increased, adding to the vast plastic and microplastic waste in the environment^4^. Furthermore, the UNEP in its fourth meeting last November 2020 reported that nearly 90 million plastic medical masks are required every month through the still on-going COVID-19, creating a new challenge for the marine plastic litter^5^. Accordingly to current trends, the total plastic produced is estimated to rise by 33 billion tons by 2050^6,7^.

The most important sources of plastic pollution in oceanic environments are coastal cities, ports, shipping activities, coastal landfills and coastal dumping sites^8,9^. Once plastic debris go into the ocean, they break down into microplastics by photolytic, mechanical and biological degradation^10^. Several studies on plastic size abundance and distribution have shown a permanent fragmentation of microplastic from larger to smaller, to nanoplastics (< 25 µm), occurring continuously in the oceans^8,11^. One of the main concerns about the smaller fraction of plastic particles is the risk potential for filter feeders, which tend to confuse it for plankton and end up consuming plastic debris^12–14^.

Microplastics ingestion has been reported in a wide range of marine organisms from different trophic levels. The increasing scientific evidence that marine organisms of human consumption ingest microplastics directly from the seawater or from lower trophic levels^8,14^, confirms that these microplastic particles have infiltrated the marine ecosystem and are currently be underestimated^15^. Plastic debris either float through the seawater column or sink when they become covered in biofilm, and settle into the sediments^6,16^. Plastic particles of all range sizes not only contain additives but also other anthropogenic contaminants, such as organic chemicals that are adsorbed from surrounding seawater^7,17^. These pollutants include persistent, bioaccumulative, and toxic substances (PBTs), such as polychlorinated biphenyls (PCBs) and dioxins. Due to the pollutants’ hydrophobicity, these contaminants have greater affinity for plastics than seawater and natural sediments^18^. Microplastics particles appear to act as carriers of these contaminants to wildlife. When ingested by marine organisms, PBTs can be released to digestive fluid and can be transferred to the tissues^19^. These chemicals can infiltrate into cells, react with important biomolecules and cause endocrine disruption^20^. In addition, plastics not only have the potential to transport contaminants, but they can also increase their environmental persistence^3^.

Laboratory experiments have showed the potential of microplastics to be transferred via planktonic organisms from one trophic level to a higher level^10,21^. This may be due to particle size range analysed, which was limited to microplastic > 150 µm, and the nanometre range has proven to have greater capacity for tissue translocation^3,22^. Accumulation of plastic micro-particles in lower trophic levels could lead to a domino effect in marine food webs^23,24^, affecting ultimately humans. This highlights the importance of plastics as a source of contaminants of emerging concern for environmental and human health.

Historically, plastic debris have been reported and documented at higher density in the Northern Hemisphere oceanic basins when compared to the Southern regions^25,26^. The highest concentrations of plastic debris reported until now are found in the central areas of the North Atlantic and North Pacific Oceans^27,28^. However, there is a clear lack of studies in many oceanic basin regions where data on plastic debris remain unknown. Additionally, oceanic circulation models suggest that all five subtropical ocean gyres act as convergent zones by Ekman currents making them the most likely accumulation regions^29^. As surface ocean currents are spatial and temporal variables, the highest concentrations of plastic debris are constantly fluctuating. However, there is limited available data on the sources and dispersion of plastic litter along the Tropical South American coast and the Galápagos archipelago^30^.

While there is limited data on the Tropical Eastern Pacific and around the multiple archipelagos of this region, there is no reason to expect that these zones remain unaffected by microplastics pollution. Thus, this study had as a goal the detection and quantification of microplastic in oceanic surface water, and marine organism of human consumption. Moreover, by using spatial design interpolation models based on marine oceanic currents, we attempted to measure the distribution and concentrations of microplastics within the study zone.

## Materials and methods

### Sampling and processing of water samples

A 25 day-expedition took place on-board the Orion vessel in October 2017, sailing across the Tropical Eastern Pacific and Galápagos archipelago covering an approximated area of 453,000 square-kilometres. The route included a 4000 km-trajectory with 40 sampling stations (see Supplementary S1 for geospatial location points). Environmental water samples were collected under permit # MAE-DNB-CM-2016-0045 in collaboration with the National Institute of Biodiversity granted by the local Ecuadorian Ministry of Environment and Water.

In order to collect the oceanic water samples, two plankton nets with a 60 cm-diameter, 3 m-length, and 150 μm- and 500 μm pore size, respectively, were used. Both nets were simultaneously launched at a distance of 30 m from the stern of the ship in order to prevent any oil or litter contamination from the main vessel. The nets were superficially dragged for a period of 5 min at each station, with a speed of 2 knots (3.70 km/h). A rough calculation using the volume flow rate formula (Q = [A × s] × t; where Q = volume flow rate, A = area, s = speed or velocity, and t = dragging time) allows estimating that, on average, at least 550,000 L (550 m^3^) of seawater were filtrated at each station. Then, the nets were picked up using a pot line hauler and washed employing a high-pressure seawater hose to collect all organic and inorganic matter into the top end of each net. Later, the content of the top end was transferred to a 500 mL glass flask, preserved in 70% ethanol and stored for further analysis. At each station, 500 mL water control samples were taken from the tube hose from ocean water pumped into the water circulation system to confirm this was not a potential source of microplastic particles contamination.

Between stations, the nets were thoroughly rinsed with ultrapure water to get rid of any residues and were let to dry to guarantee and avoid cross-contamination between samples. Back in the lab, the samples were sifted, using distilled water, into a filtration system consisting of a Glenammer sediment testing set (5000, 1000, 750, 500 and 150 μm). All the organic and inorganic particles that were trapped in test sieves were inspected and separated. Microplastic particles were classified into category sizes. Four categories for the 150 μm-plankton net: 150–500, 501–750, 751–1000 and 1001–5000 μm; and three categories for the 500 μm-plankton net: 500–750, 751–1000, and 1001–5000 μm, which were counted under a stereomicroscope. Organic particles were kept separately for further inspection of ichthyoplankton and copepods. All remaining organic and inorganic material after the last filtration with the lowest diameter test sieve (150 μm) were treated with 30%-hydrogen peroxide to get rid of organic matter^31^, and were then further filtered in a vacuum system employing 100 μm microcellulose filters (Whatman). The remaining water was stored in cold at 4 °C for any future potential analysis with more sensitive and precise technology into nanoparticles. The entire system was rinsed with ultrapure water and 70% ethanol between each sample filtration to avoid cross-contamination. Extreme care was taken to not contaminate the samples by keeping the filtration system covered and washing the transfer apparatus with ultrapure water and 70% ethanol multiple times. All washing and purification solutions were filtered through to minimize any sample loss due to adhesion of microplastics on the wall of any part of the filter apparatus. The microplastic isolation was repeated three times for each sample to ensure recovery.

The microcellulose filters were inspected in an AmScope trinocular stereoscope with digital camera, and visual counting of microplastic particles and fibres was done using millimetric background glass filter especially designed for this purpose (Petroff–Hausser counters). Filters were then inspected and a microplastic particle counting was done using a BX53 Olympus microscope. Additionally, presence of the microplastic fibres and particles were confirmed by using UV-light lamps implemented in the same microscope instrument.

### Sampling and processing of marine organisms

To analyse plastic presence in marine organisms of human consumption, 15 specimens of each of the 16 different species collected, including molluscs, fish and crustaceans were bought across the most representative market ports in all four provinces (El Oro, Santa Elena, Manabí and Esmeraldas) evaluated in the Pacific coast of Ecuador (see Supplementary S1), under the same permit mentioned above. They were preserved frozen at – 20 °C. Samples were then dissected and tissue from the digestive tract and the dorsal muscle were investigated for each specimen. The collected samples were analysed in a BX53 Olympus microscope coupled with a microscale to visually quantify the presence of microplastic particles over 200 μm.

For muscle inspection, 0.5 cm^3^-muscle tissue fragments were imbibed in paraffin. These preparations were tanned with hematoxylin and eosin (H–E) technique and cut with a microtome^32^. Tissue slices were then prepared on microscope plates using Entellan resin and inspected for microplastic presence under the BX53 Olympus microscope. The figure presenting the concentrations of microplastic particles in marine organisms was made on Adobe Acrobat DC Pro (https://acrobat.adobe.com); organism illustrations were obtained at www.pexels.com (free access and use) and manually adjusted to the figure.

### Quantification, statistical analysis and spatial interpolations

Microplastic particles were quantified and total values were determined using counting chambers of 0.2 mm × 0.2 mm centre square cover glass (Petroff–Hausser counters). Data was tabulated including the exact location of each sampling site, date and the total number of microplastic particles per station with each individual net, and by combining the total amounts from both 150 and 500 μm-plankton nets. Data was then exported to *Minitab 18.1.0.0* Statistical Software^33^ where the one-way analysis of variance (ANOVA) was performed to identify statistical differences between particles sizes and stations. The mean differences in the groups were evaluated with Fisher's LSD method with a 95% confidence interval.

In order to assess the extent of contamination (microplastics presence and distribution), the study area was divided into four zones: (A) Continental waters, (B) International waters, (C) Eastern Galapagos and (D) Western Galápagos, within the total of 40 sampled stations. A spatial interpolation analysis was performed in *ArcGIS 10.4.1* software^34^ for the collected microplastics data with combined values for the two nets. Two tools: the Topo to Raster and the Create Contours tools were mainly used. The Topo to Raster tool available in *ArcGIS* was used as the interpolation method. An oceanic photograph of free access (http://www.apollomapping.com/geoeye/satellite) was used as the first layer for the interpolation. Further layers containing the sampling points and microplastic concentrations data were later on added. A study area around the microplastic sampling locations was defined and used as boundary for the interpolation. The Create Contours tool was then used to create 1-unit (μp/m^3^) contours from the raster image produced by the interpolation tool.

By using the known concentrations of microplastic particles accounted for the combined net values, with the precise oceanic coordinates (stations), estimated values were determined at the remaining unknown points. The result is an interpolation-contours figure showing a possible scenario of the spatial distribution of the microplastics sampled in the studied area. We assumed that for any microplastic particles measured, their magnitude should be equal or greater than zero (μp/m^3^). The assumption was used to condition the limits of the interpolation method, so the produced raster image contains only numbers equal to or greater than zero.

## Results

### Seawater samples

**Figure 1:**
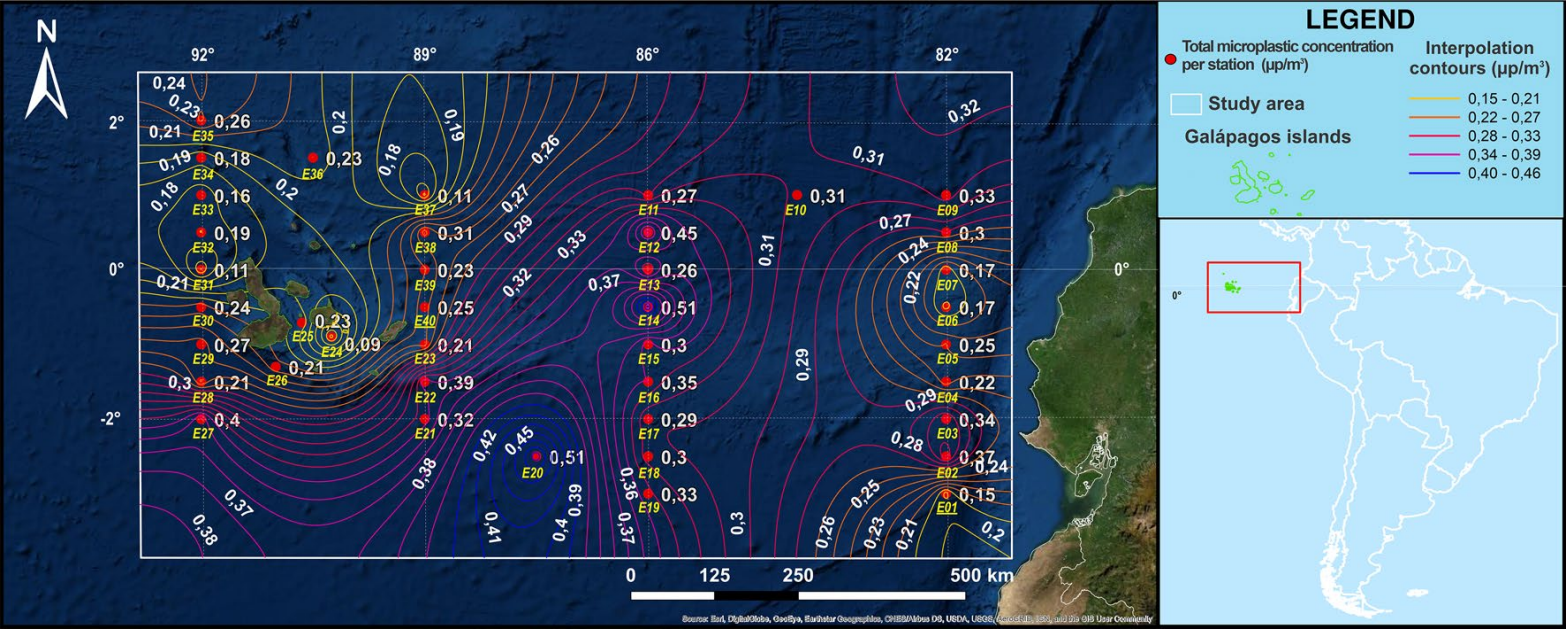
Spatial interpolation of the microplastic particle concentrations in the study area. Using the known values of microplastic particles concentrations determined (µp/m^3^), combining both 150 and 500 µm-plankton nets at the precise oceanic stations, estimate concentration values are determined at the remaining unknown spatial points. The Southeast and Northwest presented the lowest microplastics concentration, which was coloured in blue. The highest microplastic concentration was observed in international waters in the central to southern part of the study area coloured in red, potentially associated with ocean circulation patterns.

Microplastic particles were detected in 100% of the collected samples from the 40 stations across the 4000 km trajectory expedition. Moreover, microplastic particles in all size ranges were observed in 100% of the filtered samples analysed (see Fig. 1 and Supplementary S2).

The highest concentration (μp/m^3^) by particles size collected with the 150 μm-plankton net was observed for the smallest category (150–500 µm). This category concentrated 71% of the microplastic particles with 0.15 ± 0.05 (mean ± s.d., respectively) with a significant difference (*p* < 0.001) for the three size ranges. The second highest microplastic concentration was found in the category of 501–750 µm, with a 15% (0.03 ± 0.02). The range of particles within 751–1000 µm concentrated a 6% (0.01 ± 0.01). The largest particle size range (1001–5000 µm) had an overall 8% concentration of the particles per station (0.02 ± 0.01).

As for the second 500 μm-plankton net, the highest concentration by particle size was also observed for the smallest category (500–750 µm) in its class, which concentrated 45% of the particles (0.03 ± 0.01). The second highest concentration of microplastics was found in the 751–1000 µm category, with 22% prevalence (0.01 ± 0.01). The largest particle size range (1000–5000 µm) presented a 33% prevalence (0.02 ± 0.01). Furthermore, a high significant difference was detected between the three categories (*p* < *0.01*), confirming the vast amount of microplastic particles detected in the size 500–750 µm when compared with the other two larger sizes (see Supplementary S2).

Plastic concentrations were also quantified by zones. Stations within continental waters had 0.26 ± 0.08, international waters stations 0.36 ± 0.10, while 0.24 ± 0.09 and 0.22 ± 0.08 were registered for Eastern and Western Galápagos stations, respectively in μp/m^3^. Highest concentrations were detected within international waters. The one-way ANOVA test (see Supplementary S3) revealed a statistically significant difference between the four sub-regional zones (*p* < 0.01).

**Figure 2:**
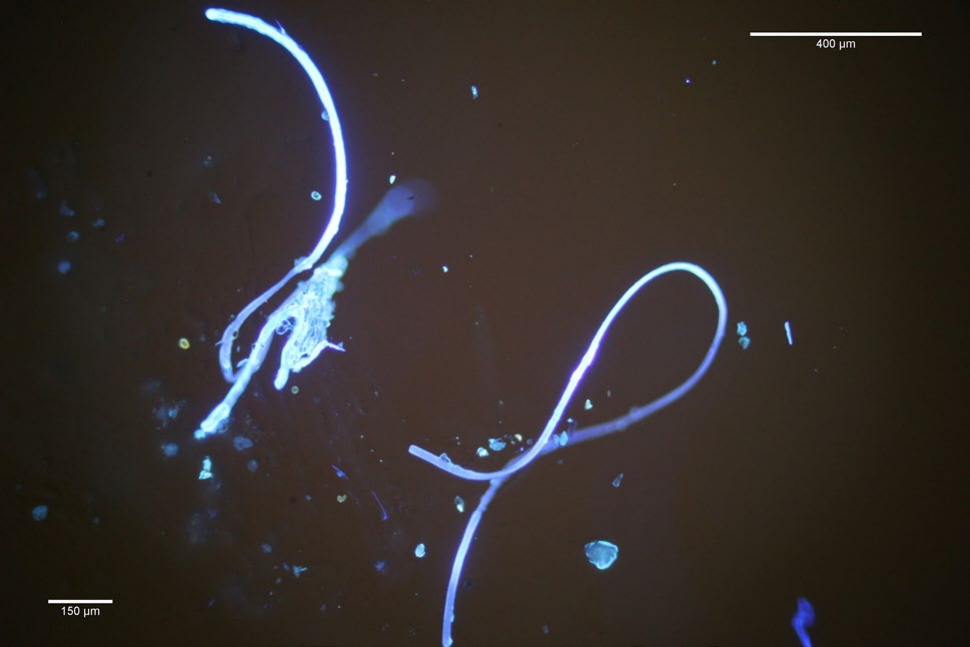
Microplastic fibres and particles under the microscope. Each of the filters collected was inspected and investigated under the microscope to quantify the amount of microplastic fibres and particles. Most polymers, the main structural molecular blocks of plastics, tend to shine under the ultraviolet light (UV-light), which was done using a BX53 Olympus microscope.

Microplastics appeared mostly in the form of plastic fibres (see Fig. 2), which were found in all collected samples. As mentioned above, the largest concentration of microplastic particles was found in international waters at the station 20 (see Supplementary S3 and Fig. 1).

### Marine organisms

**Figure 3:**
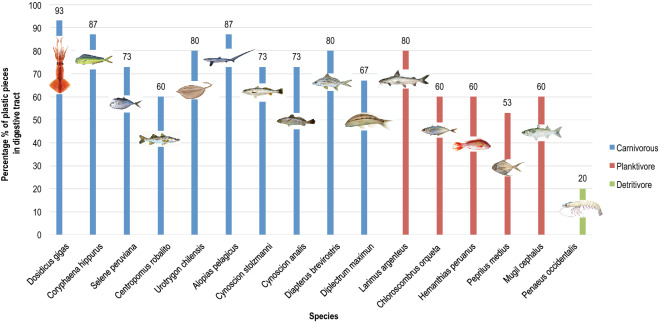
Prevalence of microplastic particles in the digestive track of marine species. Microplastic particles found in 16 different marine species of human consumption that were bought in the most representative ports in all four provinces (Manabí, El Oro, Esmeraldas and Santa Elena) in the Pacific coast of Ecuador were quantified. Marine organisms were categorized by their feeding behaviour: carnivorous, planktivory, and detritivore. Fifteen specimens (n = 15) were taken per each of the 16 species analysed.

A total of 16 species were analysed and clustered by their feeding behaviour, finding the highest microplastic prevalence in carnivorous species, while animals that feed from dead organic matter (detritivore species) were found with the lowest. We investigated microplastic particles in the digestive tracts and muscle tissue of 240 marine organisms of human consumption including fish (210 specimens: 15 of each of 14 species), cephalopod molluscs (15 specimens of one species) and crustaceans (15 specimens of one species). Plastic fragments over 200 μm were detected in the digestive tract of 166 out of 240 specimens (69%) from the 16 different species analysed. Microplastics were found in 149 (71%) of 210 fish from the 14 different species (see Fig. 3). In overall, 77% of the carnivorous species presented microplastic pieces in their digestive tract, followed by planktivorous (63%) and detritivore (20%). No plastic was found in muscle tissues.

### Spatial interpolations

The graph (see Fig. 1) indicates that the regions in the Southeast (82° longitude) near the continental coast (0.26 ± 0.10) and Northwest (92° longitude) in the Galápagos (0.22 ± 0.09) presented the lowest microplastics concentration, with the addition of a small region in between islands (stations E22–E27). The highest microplastic concentration was observed in international waters (0.36 ± 0.09) in the central to southern part (stations E11–E20) with the highest recorded concentration of the study area at station E20 with a 0.51 μp/m^3^.

## Discussion

### Microplastic particles in ocean water

Plastic pollution in the oceans is directly correlated with this material being robust and durable, which is linked to the high amounts of plastics produced, used and easily discarded^1,35^. Microplastic fragments have been found in sedimentary habitats, shores, pelagic zones^7,36^, deep sea^37^ and in living organisms^38^, including humans^14^. Worldwide production and uncontrolled disposal of face masks and many other medical-health supplies have dramatically increased during the current COVID-19 pandemic, creating a vast new challenge for plastic litter entering the environment. While governments and international organizations work together to find solutions to reduce the amount of all residual plastic waste, delaying action by 5 years could increase plastic pollution in the oceans by around 80 million metric tons^5^.

In our study, microplastic particles in all the four range sizes analysed (150–500, 501–750, 751–1000 and 1001–5000 µm) had a 100% prevalence across all stations (see Supplementary S2). The size distribution of plastic particles in the seawater samples showed that the smallest size class, between 150 and 500 µm, is more abundant than the larger sizes. Other authors have also reported that smaller microplastic sizes abundance is a common characteristic result of the plastic size distribution among the oceans^19,39^. In addition, several studies on microplastic size abundance and distribution have shown a permanent fragmentation of microplastic from larger to smaller, to even into nanoplastic (< 25 µm), occurring continuously in the oceans^8^, and in all aquatic environments^16^. The main global concern about the predominance of this size class is its risk potential for filter feeders, which tend to confuse it for plankton and end up consuming plastic debris^14^. Further analytical chemistry characterization of the polymers type and POP’s present in the samples at each station, was originally intended in this study to cover the smaller fraction and nanoplastic molecular classification. Nevertheless, molecular oil residues were detected to cause contamination in the samples, which unable this analysis to be implemented to confirm the characterization of polymers and POP’s.

### Interpolation of microplastic concentrations

Oceanic circulation models suggest the highest concentrations of plastic debris are accumulated along the five main subtropical ocean gyres defined as convergent zones by the Ekman currents^25,40^. As such, ocean currents play a major role in the origin source, transportation, distribution and accumulation of plastic debris around the world. In our study area, several station points were detected with large concentrations of microplastic particles mostly in the central to southern part of the study area, which were outside the local small gyres present (see Fig. 1). These findings are coherent with basin-scale microplastic particles transport that explain sources and pathways of microplastic that end up in the Galápagos Archipelago^30^ from far South oceanic basins. However, Costa Rica and other countries farther north can be considered as plastic particle origin sources if simulations are not limited to surface currents.

Plastic transport may also depend on the sinking processes that plastic particles undergo when they reach the ocean^6,28^. Microplastics show different buoyancy characteristics depending on the plastic polymers and additives they are made of^41^. Around 60% of all plastic items produced are less dense than seawater^3^. Biofouling and other interactions with marine biota, degradation, fragmentation or additives leaching may accelerate the sinking process of derived plastic particles^30^. The impact of microplastics in the marine environments, however, depends on physical behaviours (migration, sedimentation and accumulation), chemical behaviours (degradation and adsorption) and bio-behaviours (ingestion, translocation and biodegradation)^18^. Still, trawl sampling efforts coupled with vessel-based sighting surveys confirm that available data on quantities and characteristics of buoyant plastic particles in the nanoplastic range represent only 13% of the available buoyant plastic mass^1^. Therefore, new insights have coupled measured concentrations of ocean plastic of different sizes and types, dispersal models, geo-referenced imaginary and seasonal and intern annual changes to improve the estimations of plastic debris in the upper water column^7^.

The Galápagos archipelago and its Marine Reserve lay 1000 km off the coast of the South American coastline and are among the most emblematic wildlife refuges in the world. However, plastic litter and microplastic residues have recently been found even in this isolate group of islands and around its waters. To our knowledge, prior to this study, the levels of this microplastic contamination and its quantification on Galápagos coastlines and across the Eastern Tropical Pacific were barely known and limited to one single study^30^.

### Microplastic in marine organism of human consumption

Plastic particles in the digestive systems of many species of fish and other marine organisms consumable by humans have been reported and quantified^23,42^. Recent studies on plastic size abundance and distribution have shown a continuous fragmentation of microplastic into nanoplastic occurring constantly in the oceans by marine organisms ingesting microplastics and bio-accumulating these particles in their stomachs^2,3^.

In the present study, microplastic contamination and consumption by marine organisms were reported through the quantification of microplastic particles in the digestive tract of 240 marine organisms of human consumption including fish, cephalopod molluscs and crustaceans (see Fig. 3). Microplastic fragments were detected in 166 out of 240 specimens (69%) from the 16 different species analysed. Moreover, microplastic particles were found in 149 (71%) of 210 fish from 14 different species (in at least eight specimens for each of all fish species analysed). This value is higher than those previously reported^43^, which allows to conclude that microplastic debris in the form of fish feed, may accumulate over time and space. We suspect that this value may have considerably increased during the last year (2020-2021) as a direct consequence of the massive plastic litter produced and discarded into the environment through the COVID-19 pandemic.

Among all different species analysed in this work, 77% of the carnivorous species presented microplastic pieces in their digestive tract, followed by planktivorous (63%) and detritivores (20%). As previously stated, contamination of microplastic particles of all sizes in the Oceans are easily mistaken with food by marine organisms, especially when they overlap with the size range of their prey^9^. From the total 16 examined species, *Dosidicus gigas,* commonly known as giant squid, reached 93% microplastic prevalence in its digestive tract. It was followed by *Alopias pelagicus* and *Coryphaena hippurus* with 87% prevalence each. All three are carnivorous species. In a previous study, plastic ingestion in carnivorous species of fish^42^ ranged from < 1 to 58%. The 77% obtained in our research breaks the normal parameters, showing that tropical Pacific Equator coast has worrying high levels of microplastic pollution in comparison with reports from other Pacific oceanic basins.

On the other hand, planktivorous species are thought to develop mechanisms to avoid consuming microplastic particles^44,45^. It has been suggested that planktivorous fish species may have a low risk of plastic ingestion in superficial waters^27^. Yet, the 63% prevalence in planktivorous fish analysed in the present work is considerably high when compared to the 5% prevalence found from a previous study^26^.

In spite of scientific evidence of plastic entrance to different tissues than those related to the digestive tract^41,46^, no plastic was found in the muscle tissue from the 240 marine organisms examined in this study. This may be due to particle size range analysed, which was limited to microplastic > 150 µm, and the nanometre range has proven to have greater capacity for tissue translocation^3,22^. As briefly mentioned above, our research originally planned to do an analytical chemistry characterization of the type polymers and POP’s present in the samples at each station. Special filter samples were simultaneously collected at each station for this purpose. However, molecular oil residues contamination was detected in all analysed samples in the lab, which unable to achieve this complementary part of the study and thus, this entire section was excluded from the manuscript. As of today, the cause of the oil contamination remains unknown.

To the best of our knowledge, this is one of the first systematic reports visually quantifying microplastic abundance and modelling its distribution across a section of the Tropical Eastern Pacific and around the Galápagos archipelago. Finally, this is also the first time that microplastic particles are detected and quantified in marine organisms of human consumption in this region.

## Supplementary Information

Supplementary Information 1.

Supplementary Information 2.

Supplementary Information 3.
