# Author Correction: Microplastic pollution in seawater and marine organisms across the Tropical Eastern Pacific and Galápagos

**DOI:** 10.1038/s41598-022-07504-w

**Published:** 2022-02-25

**Authors:** Alonzo Alfaro-Núñez, Diana Astorga, Lenin Cáceres-Farías, Lisandra Bastidas, Cynthia Soto Villegas, Kewrin Choez Macay, Jan H. Christensen

**Affiliations:** 1 Virus Research & Development Laboratory, Statens Serum Institut, Artillerivej 5, 2300 København S, Denmark; 2 Section for Evolutionary Genomics, GLOBE Institute, University of Copenhagen, Øster Farimagsgade 5, 1353 Copenhagen K, Denmark; 3 Escuela de Ciencias Biológicas, Pontificia Universidad Católica del Ecuador, Av. 12 de Octubre 1076, 17-01-2184 Apartado, Quito Ecuador; 4 Grupo de Investigación en Sanidad Acuícola, Inocuidad y Salud Ambiental, Escuela de Acuicultura y Pesquería, Facultad de Ciencias Veterinarias, Universidad Técnica de Manabí, Ciudadela Universitaria, Leonidas Plaza EC131402, Bahía de Caráquez, Ecuador; 5 AquaCEAL Corp. Urb, Las Palmeras, av. Capitán Byron Palacios y General Quisquis, #8 EC230101, Santo Domingo de los Colorados, Ecuador; 6 School of Biological Science and Engineering, Yachay Tech University, Urcuquí, Imbabura Ecuador; 7 Department of Plant and Environmental Sciences, University of Copenhagen, Thorvaldsensvej 40, 1871 Frederiksberg, Denmark

Correction to: *Scientific Reports* 10.1038/s41598-021-85939-3, published on 19 March 2021

The original version of this Article contained errors.

The negative blank control data was omitted from the analyses described in the paper. This is now corrected in the Methods, where the following sentence was added at the end of 'Sampling and processing of water samples',

"Negative blank controls were collected, inspected, and quantified at each station after rinsing the filtration system as previously described."

Statistical analysis of this data is now also included in a newly added Supplementary File 4. In Results, under 'Seawater samples',

"Further- more, a high significant difference was detected between the three categories (p < 0.01), confirming the vast amount of microplastic particles detected in the size 500–750 μm when compared with the other two larger sizes (see Supplementary S2)."

now reads:

"Further- more, a high significant difference was detected between the three categories (p < 0.01), confirming the vast amount of microplastic particles detected in the size 500–750 μm when compared with the other two larger sizes (see Supplementary S2 and S4)."

and,

"The one-way ANOVA test (see Supplementary S3) revealed a statistically significant difference between the four sub-regional zones (p < 0.01)."

now reads:

"The one-way ANOVA test (see Supplementary S3 and S4) revealed a statistically significant difference between the four sub-regional zones (p < 0.01)."

Additionally,

“Microplastics appeared mostly in the form of plastic fibres (see Fig. 2), which were found in all collected samples. As mentioned above, the largest concentration of microplastic particles was found in international waters at the station 20 (see Supplementary S3 and Fig. 1)."

now reads:

"Microplastics appeared mostly in the form of plastic fibres (see Fig. 2), which were found in all collected samples. As mentioned above, the largest concentration of microplastic particles was found in international waters at the station 20 (see Supplementary S3, S4 and Fig. 1)."

Furthermore, name of the sixth author, Kewrin Macay, was incorrectly listed. It now reads Kewrin Choez Macay.

The original Article and accompanying Supplementary Information files have been corrected.

