# Supplementary material for: Microplastic pollution in seawater and marine organisms across the Tropical Eastern Pacific and Galápagos
Source: Sci Rep. 2021 Mar 19;11:6424. doi: 10.1038/s41598-021-85939-3 (PMC7979831; doi:10.1038/s41598-021-85939-3)

S1.

Title page

Title

Microplastic pollution in seawater and marine organisms across the Tropical Eastern Pacific and Galápagos

Authors

Alonzo Alfaro-Núñez*, Lenin Cáceres-Farías, Lisandra Bastidas, Cynthia Soto Villegas and Diana Astorga

* **Corresponding author**: alonzoalfaro@gmail.com, alnz@ssi.dk

S1. Geospatial location points of the 40 sampling stations. For the 4,000 km trajectory across the Tropical Eastern Pacific and Galápagos archipelago, the exact geospatial location of the 40 stations where water samples were collected.

Stations	Latitudinal degrees	Longitudinal degrees
Salinas	-2,160	-81,001
E-01	-3,00	-82,00
E-02	-2,50	-82,00
E-03	-2,00	-82,00
E-04	-1,50	-82,00
E-05	-1,00	-82,00
E-06	-0,50	-82,00
E-07	0,00	-82,00
E-08	0,50	-82,00
E-09	1,00	-82,00
E-10	1,00	-84,00
E-11	1,00	-86,00
E-12	0,50	-86,00
E-13	0,00	-86,00
E-14	-0,50	-86,00
E-15	-1,00	-86,00
E-16	-1,50	-86,00
E-17	-2,00	-86,00
E-18	-2,50	-86,00
E-19	-3,00	-86,00
E-20	-2,50	-87,50
E-21	-2,00	-89,00
E-22	-1,50	-89,00
E-23	-1,00	-89,00
E-24	-0,90	-90,25
E-25	-0,71	-90,65
E-26	-1,30	-91,00
E-27	-2,00	-92,00
E-28	-1,50	-92,00
E-29	-1,00	-92,00
E-30	-0,50	-92,00
E-31	0,00	-92,00
E-32	0,50	-92,00
E-33	1,00	-92,00
E-34	1,50	-92,00
E-35	2,00	-92,00
E-36	1,50	-90,50
E-37	1,00	-89,00
E-38	0,50	-89,00
E-39	0,00	-89,00
E-40	-0,50	-89,00

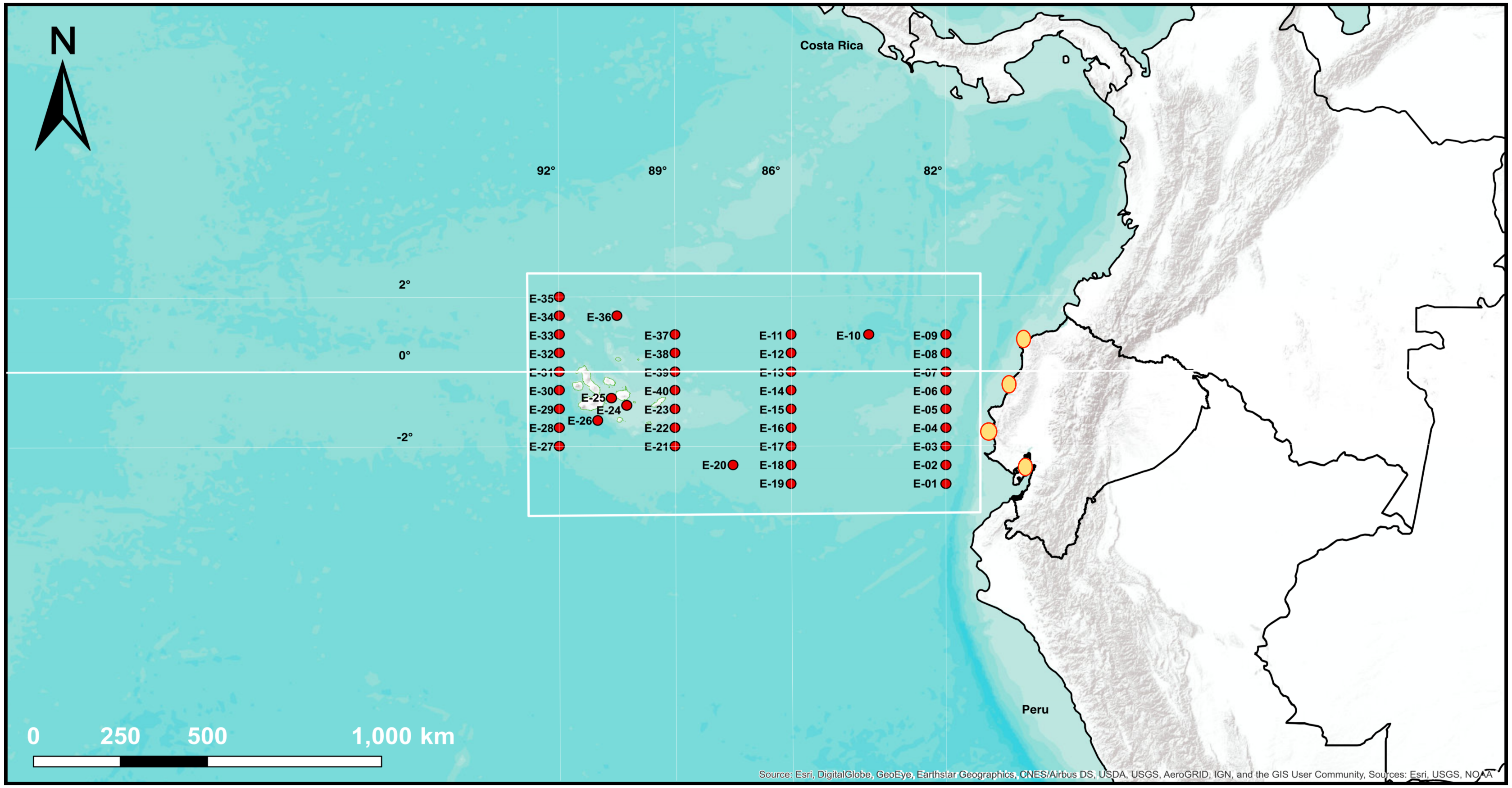

Supplement: Supplementary file 1 — Supplementary Information 1. [file 41598_2021_85939_MOESM1_ESM.pdf]
