# Supplementary material for: Microplastic pollution in seawater and marine organisms across the Tropical Eastern Pacific and Galápagos
Source: Sci Rep. 2021 Mar 19;11:6424. doi: 10.1038/s41598-021-85939-3 (PMC7979831; doi:10.1038/s41598-021-85939-3)
Supplement: Supplementary file 2 — Supplementary Information 2. [file 41598_2021_85939_MOESM2_ESM.pdf]

S2.

Title page

Title

Microplastic pollution in seawater and marine organisms across the Tropical Eastern Pacific and Galápagos

Authors

Alonzo Alfaro-Núñez*, Lenin Cáceres-Farías, Lisandra Bastidas, Cynthia Soto Villegas and Diana Astorga

* **Corresponding author**: alonzoalfaro@gmail.com, alnz@ssi.dk

S2. Microplastic particles by size in the Ocean

Geographic location		Plankton net 150 µm					Plankton net 500 µm			
Zone	Stations	150 - 500 µm	501 - 750 µm	751 - 1000 µm	1001 - 5000 µm	Total	501 - 750 µm	751 – 1000 µm	1001 - 5000 µm	Total
Continental	1	0.10	0.02	0.01	0.00	0.13	0.01	0.00	0.00	0.02
	2	0.24	0.03	0.02	0.01	0.31	0.03	0.01	0.02	0.06
	3	0.18	0.03	0.02	0.03	0.26	0.03	0.01	0.03	0.08
	4	0.13	0.02	0.01	0.01	0.18	0.01	0.01	0.02	0.04
	5	0.15	0.02	0.01	0.02	0.20	0.02	0.01	0.02	0.05
	6	0.10	0.03	0.01	0.01	0.15	0.00	0.01	0.01	0.02
	7	0.09	0.02	0.01	0.02	0.13	0.01	0.01	0.01	0.04
	8	0.16	0.06	0.01	0.02	0.25	0.03	0.01	0.02	0.06
	9	0.21	0.03	0.02	0.00	0.27	0.03	0.01	0.02	0.06
	10	0.17	0.04	0.01	0.03	0.24	0.03	0.01	0.03	0.07
International	11	0.14	0.03	0.01	0.02	0.20	0.03	0.02	0.02	0.07
	12	0.23	0.05	0.02	0.03	0.34	0.05	0.03	0.03	0.10
	13	0.13	0.04	0.02	0.01	0.19	0.04	0.01	0.01	0.07
	14	0.29	0.06	0.01	0.03	0.39	0.05	0.03	0.04	0.12
	15	0.16	0.04	0.02	0.01	0.23	0.04	0.01	0.02	0.07
	16	0.20	0.02	0.02	0.01	0.25	0.04	0.03	0.03	0.10
	17	0.16	0.04	0.02	0.02	0.24	0.02	0.01	0.02	0.06
	18	0.16	0.03	0.03	0.02	0.23	0.03	0.02	0.03	0.07
	19	0.18	0.03	0.01	0.03	0.24	0.04	0.02	0.02	0.08
	20	0.26	0.07	0.03	0.03	0.39	0.06	0.03	0.04	0.12
Eastern Galápagos	21	0.16	0.05	0.01	0.02	0.24	0.04	0.02	0.02	0.07
	22	0.19	0.06	0.02	0.02	0.29	0.05	0.02	0.02	0.10
	23	0.11	0.02	0.01	0.02	0.15	0.02	0.01	0.02	0.05
	24	0.06	0.00	0.01	0.01	0.08	0.01	0.01	0.01	0.02
	25	0.13	0.04	0.01	0.01	0.18	0.02	0.01	0.01	0.05
Western Galápagos	26	0.11	0.03	0.00	0.01	0.15	0.03	0.02	0.02	0.06
	27	0.22	0.03	0.02	0.03	0.30	0.04	0.02	0.03	0.10
	28	0.11	0.02	0.01	0.02	0.17	0.02	0.01	0.02	0.05
	29	0.14	0.04	0.01	0.02	0.22	0.03	0.01	0.02	0.06
	30	0.13	0.03	0.01	0.02	0.18	0.03	0.01	0.02	0.06
	31	0.06	0.01	0.01	0.01	0.08	0.01	0.00	0.01	0.02
	32	0.10	0.02	0.01	0.01	0.14	0.02	0.01	0.02	0.05
	33	0.09	0.02	0.01	0.01	0.13	0.01	0.01	0.01	0.03
	34	0.11	0.02	0.01	0.00	0.15	0.02	0.01	0.01	0.03
	35	0.15	0.02	0.02	0.02	0.21	0.02	0.01	0.02	0.05
Eastern Galápagos	36	0.13	0.03	0.01	0.01	0.18	0.03	0.01	0.02	0.05
	37	0.06	0.02	0.00	0.01	0.09	0.01	0.00	0.01	0.02
	38	0.16	0.05	0.01	0.02	0.24	0.03	0.02	0.02	0.07
	39	0.13	0.04	0.01	0.02	0.19	0.01	0.01	0.02	0.04
	40	0.14	0.02	0.01	0.02	0.19	0.03	0.02	0.02	0.07

Plankton net 150 µm

One-way ANOVA: 150 - 500 µm. 501 - 750 µm. 751 - 1000 µm. 1001 - 5000 µm

Method

Null hypothesis	All means are equal
Alternative hypothesis	Not all means are equal

Significance level α = 0.05
Equal variances were assumed for the analysis.

Factor Information

Factor	Levels	Values
Factor	4	150 - 500 µm. 501 - 750 µm. 751 - 1000 µm. 1001 - 5000 µm

Analysis of Variance

Source	DF	Adj SS	Adj MS	F-Value	P-Value
Factor	3	0.4950	0.165004	211.20	0.000
Error	156	0.1219	0,000781		
Total	159	0.6169			

Model Summary

S	R-sq	R-sq(adj)	R-sq(pred)
0.0279515	80.24%	79.86%	79.22%

Means

Factor	N	Mean	StDev	95% CI
150 - 500 µm	40	0.14805	0.05285	(0.13932. 0.15678)
501 - 750 µm	40	0.03173	0.01521	(0.02300. 0.04046)
751 - 1000 µm	40	0.013318	0.006000	(0.004588. 0.022048)
1001 - 5000 µm	40	0.01673	0.00803	(0.00800. 0.02546)

Pooled StDev = 0.0279515

Fisher Pairwise Comparisons

Grouping Information Using the Fisher LSD Method and 95% Confidence

Factor	N	Mean	Grouping		
150 - 500 µm	40	0.14805	A		
501 - 750 µm	40	0.03173		B	
1001 - 5000 µm	40	0.01673			C
751 - 1000 µm	40	0.013318			C

Means that do not share a letter are significantly different.

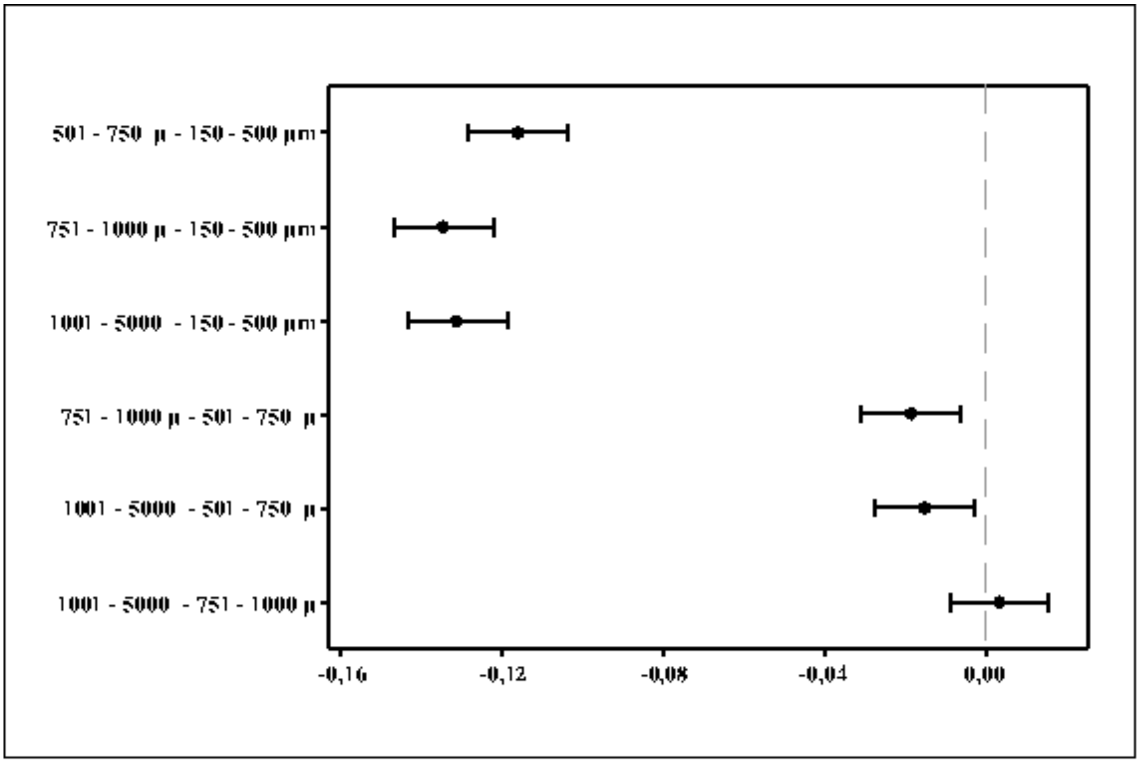
501 - 750 µ - 150 - 500 µm
751 - 1000 µ - 150 - 500 µm
1001 - 5000 - 150 - 500 µm
751 - 1000 µ - 501 - 750 µ
1001 - 5000 - 501 - 750 µ
1001 - 5000 - 751 - 1000 µ
-0,16
-0,12
-0,08
-0,04
0,00

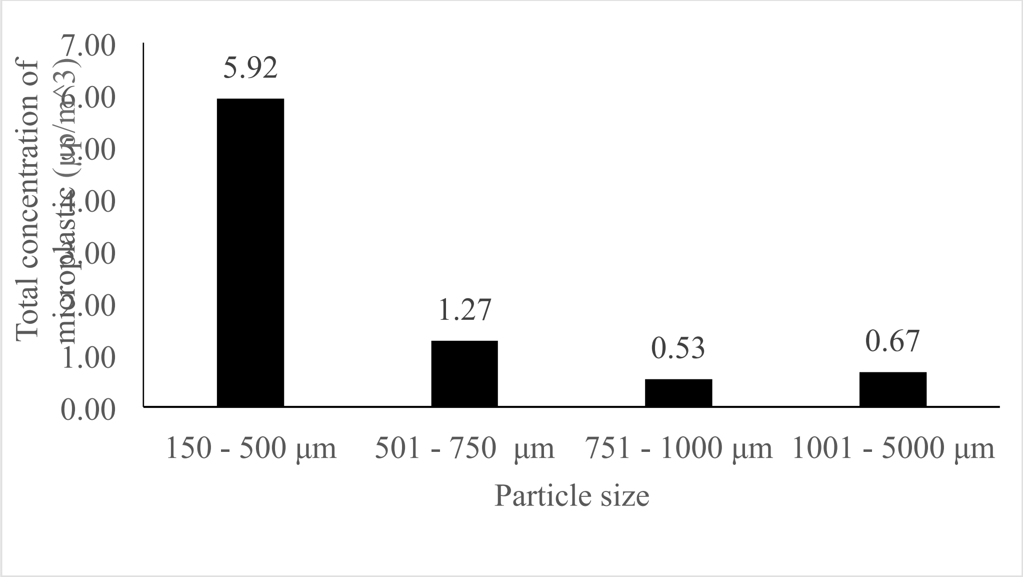
Total concentration of microplastic (µp/m^3)
7.00
6.00
5.00
4.00
3.00
2.00
1.00
0.00
5.92
1.27
0.53
0.67
150 - 500 µm
501 - 750 µm
751 - 1000 µm
1001 - 5000 µm
Particle size

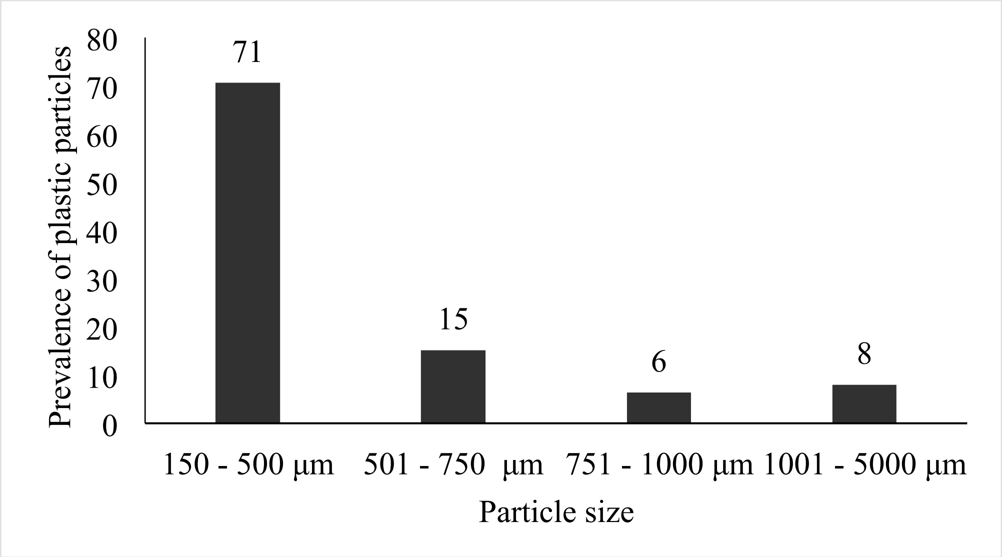

Plankton net 500 µm

One-way ANOVA: 501 - 750 µm. 751 - 1000 µm. 1001 - 5000 µm

Method

Null hypothesis All means are equal
Alternative hypothesis Not all means are equal
Significance level α = 0.05
Equal variances were assumed for the analysis.

Factor Information

Factor	Levels	Values
Factor	3	501 - 750 µm. 751 - 1000 µm. 1001 - 5000 µm

Analysis of Variance

Source	DF	Adj SS	Adj MS	F-Value	P-Value
Factor	2	0,003959	0,001979	20.43	0,000
Error	117	0,011337	0,000097		
Total	119	0,015296			

Model Summary

S	R-sq	R-sq(adj)	R-sq(pred)
0.0098437	25.88%	24.61%	22.03%

Means

Factor	N	Mean	StDev	95% CI

501 - 750 µm	40	0.02727	0.01339	(0.02419. 0.03036)
751 - 1000 µm	40	0.01323	0.00646	(0.01014. 0.01631)
1001 - 5000 µm	40	0.01955	0.00835	(0.01646. 0.02263)

Pooled StDev = 0.00984370

Fisher Pairwise Comparisons

Grouping Information Using the Fisher LSD Method and 95% Confidence

Factor	N	Mean	Grouping		
501 - 750 µm	40	0.02727	A		
1001 - 5000 µm	40	0.01955		B	
751 - 1000 µm	40	0.01323			C

Means that do not share a letter are significantly different.

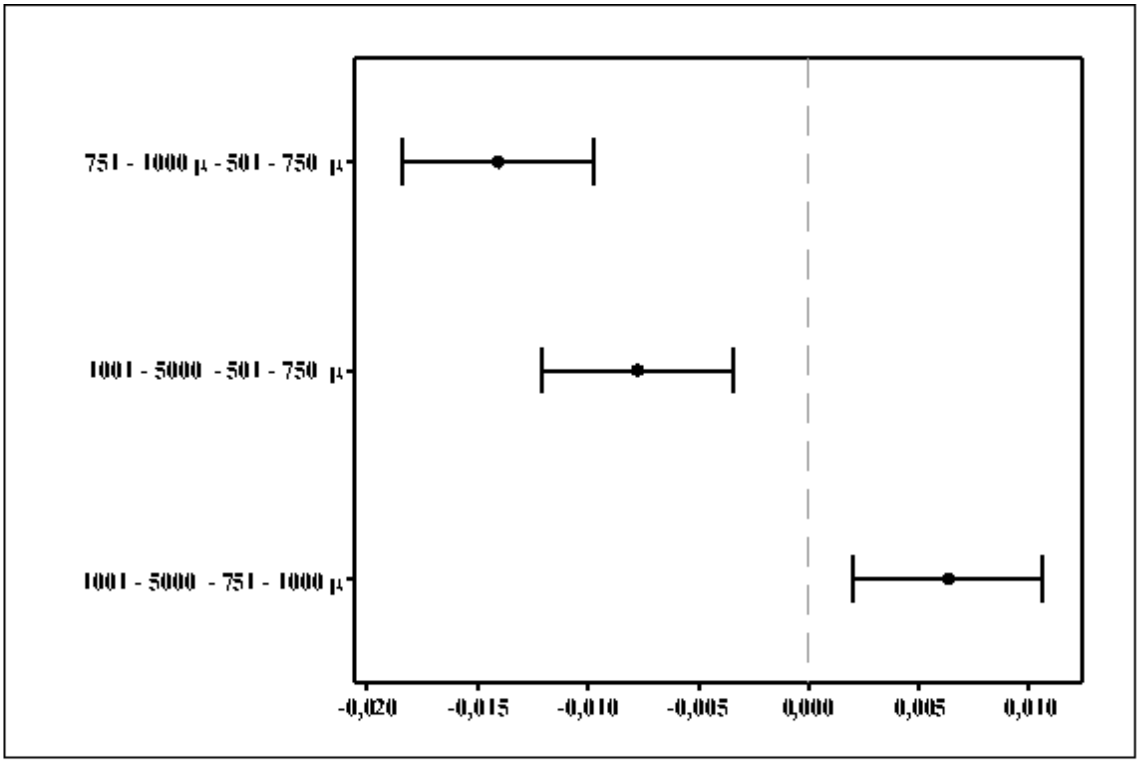

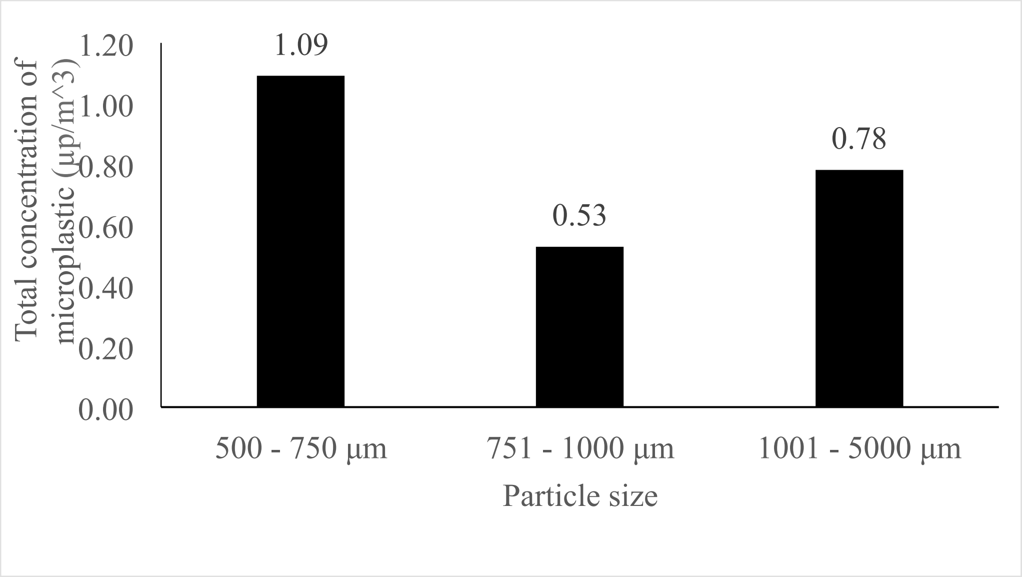
Total concentration of microplastic (µp/m^3)
1.20
1.00
0.80
0.60
0.40
0.20
0.00
1.09
0.53
0.78
500 - 750 µm
751 - 1000 µm
1001 - 5000 µm
Particle size

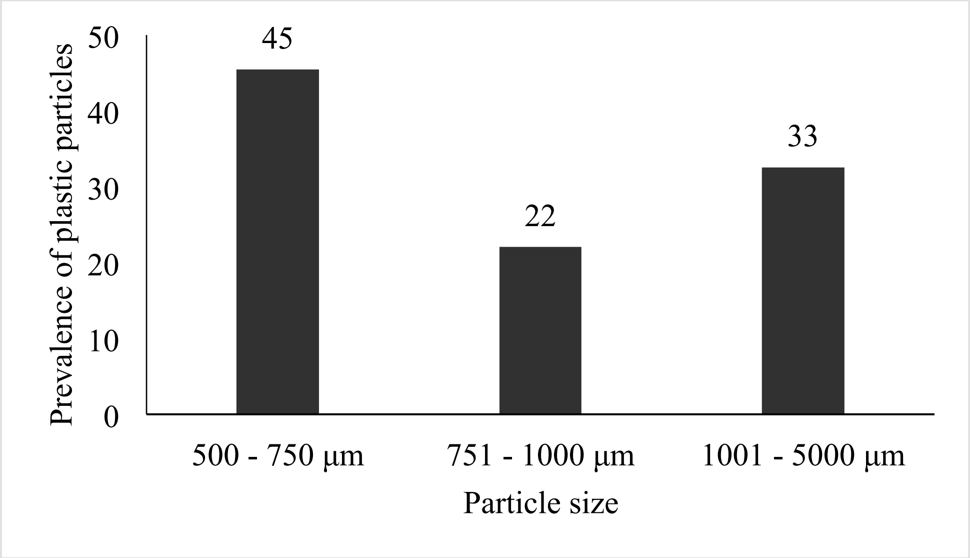
Prevalence of plastic particles
50
40
30
20
10
0
45
22
33
500 - 750 µm
751 - 1000 µm
1001 - 5000 µm
Particle size
