# Supplementary material for: Microplastic pollution in seawater and marine organisms across the Tropical Eastern Pacific and Galápagos
Source: Sci Rep. 2021 Mar 19;11:6424. doi: 10.1038/s41598-021-85939-3 (PMC7979831; doi:10.1038/s41598-021-85939-3)
Supplement: Supplementary file 3 — Supplementary Information 3. [file 41598_2021_85939_MOESM3_ESM.pdf]

S3.

Title page

Title

Microplastic pollution in seawater and marine organisms across the Tropical Eastern Pacific and Galápagos

Authors

Alonzo Alfaro-Núñez*, Lenin Cáceres-Farías, Lisandra Bastidas, Cynthia Soto Villegas and Diana Astorga

* **Corresponding author**: alonzoalfaro@gmail.com, alnz@ssi.dk

S3.

A. Total concentration of microplastic particles per station

Number stations	(μp/m^3)
1	0,15
2	0,37
3	0,34
4	0,22
5	0,25
6	0,17
7	0,17
8	0,30
9	0,33
10	0,31
11	0,27
12	0,45
13	0,26
14	0,51
15	0,30
16	0,35
17	0,29
18	0,30
19	0,33
20	0,51
21	0,32
22	0,39
23	0,21
24	0,09
25	0,23
26	0,21
27	0,40
28	0,21
29	0,27
30	0,24
31	0,11
32	0,19
33	0,16
34	0,18
35	0,26
36	0,23
37	0,11
38	0,31

39	0,23
40	0,25

**B. Concentration of microplastic particles per station 150 µm-plankton net**	
Number stations	(µp/m^3)
1	0,13
2	0,31
3	0,26
4	0,18
5	0,20
6	0,15
7	0,13
8	0,25
9	0,27
10	0,24
11	0,20
12	0,34
13	0,19
14	0,39
15	0,23
16	0,25
17	0,24
18	0,23
19	0,24
20	0,39
21	0,24
22	0,29
23	0,15
24	0,08
25	0,18
26	0,15
27	0,30
28	0,17
29	0,22
30	0,18
31	0,08
32	0,14
33	0,13
34	0,15

35	0,21
36	0,18
37	0,09
38	0,24
39	0,19
40	0,19

**Concentration of microplastic particles per station 500 µm-plankton net**	
Number stations	(µp/m^3)
1	0,02
2	0,06
3	0,08
4	0,04
5	0,05
6	0,02
7	0,04
8	0,06
9	0,06
10	0,07
11	0,07
12	0,10
13	0,07
14	0,12
15	0,07
16	0,10
17	0,06
18	0,07
19	0,08
20	0,12
21	0,07
22	0,10
23	0,05
24	0,02
25	0,05
26	0,06
27	0,10
28	0,05

29	0,06
30	0,06
31	0,02
32	0,05
33	0,03
34	0,03
35	0,05
36	0,05
37	0,02
38	0,07
39	0,04
40	0,07

Plankton net 150 µm

One-way ANOVA: Continental waters, International waters, Eastern Galapagos, Western Galápagos

Method

Null hypothesis	All means are equal
Alternative hypothesis	Not all means are equal
Significance level	α = 0.05

Equal variances were assumed for the analysis.

Factor Information

Factor	Levels	Values
Factor	4	Continental waters, International waters, Eastern Galapagos, Western Galápagos

Analysis of Variance

Source	DF	Adj SS	Adj MS	F-Value	P-Value
Factor	3	0,05941	0,019804	4,56	0,008
Error	36	0,15638	0,004344		
Total	39	0,21579			

Model Summary

S	R-sq	R-sq(adj)	R-sq(pred)
0,0659085	27,53%	21,49%	10,53%

Means

Factor	N	Mean	StDev	95% CI
Continental waters	10	0,2116	0,0626	(0,1694. 0,2539)
International waters	10	0,2718	0,0738	(0,2295. 0,3141)
Eastern Galapagos	10	0,1833	0,0663	(0,1410. 0,2255)
Western Galápagos	10	0,1725	0,0602	(0,1303. 0,2148)

Pooled StDev = 0,0659085

Fisher Pairwise Comparisons

Grouping Information Using the Fisher LSD Method and 95% Confidence

Factor	N	Mean	Grouping	
Continental waters	10	0,2718	A	
International waters	10	0,2116		B
Eastern Galapagos	10	0,1833		B
Western Galápagos	10	0,1725		B

Means that do not share a letter are significantly different.

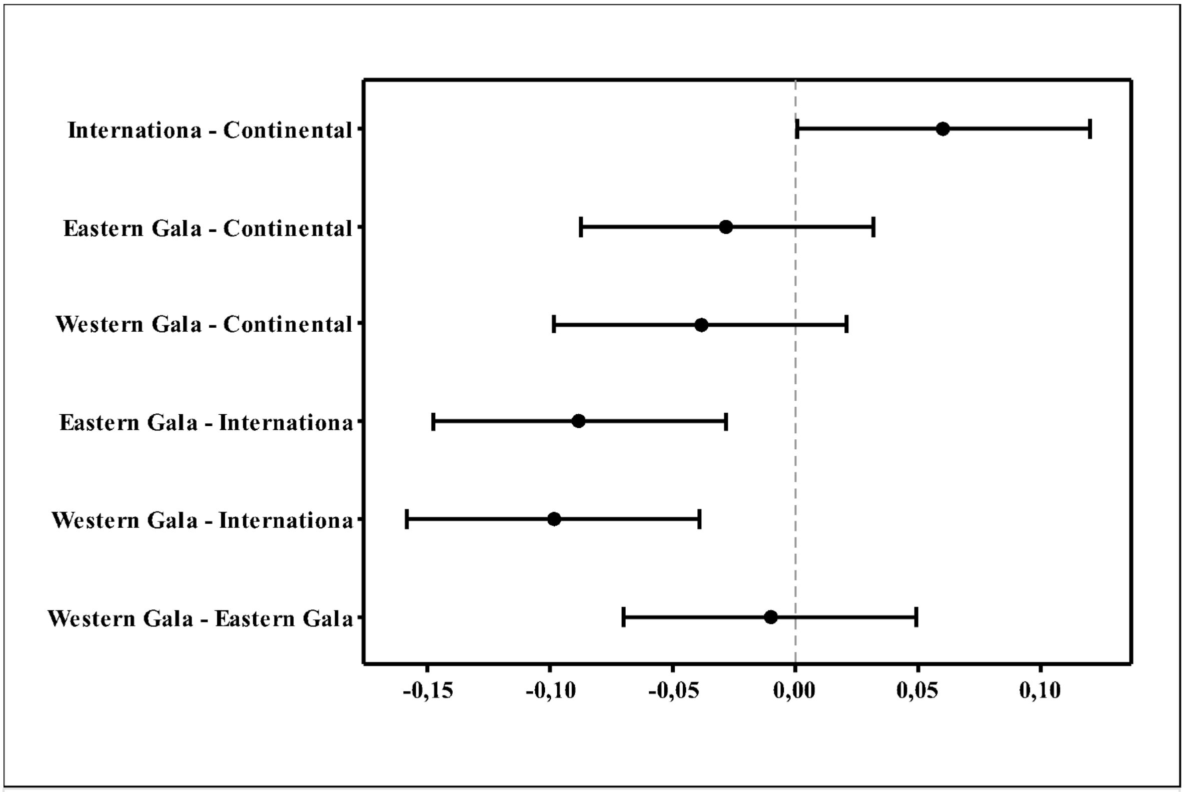

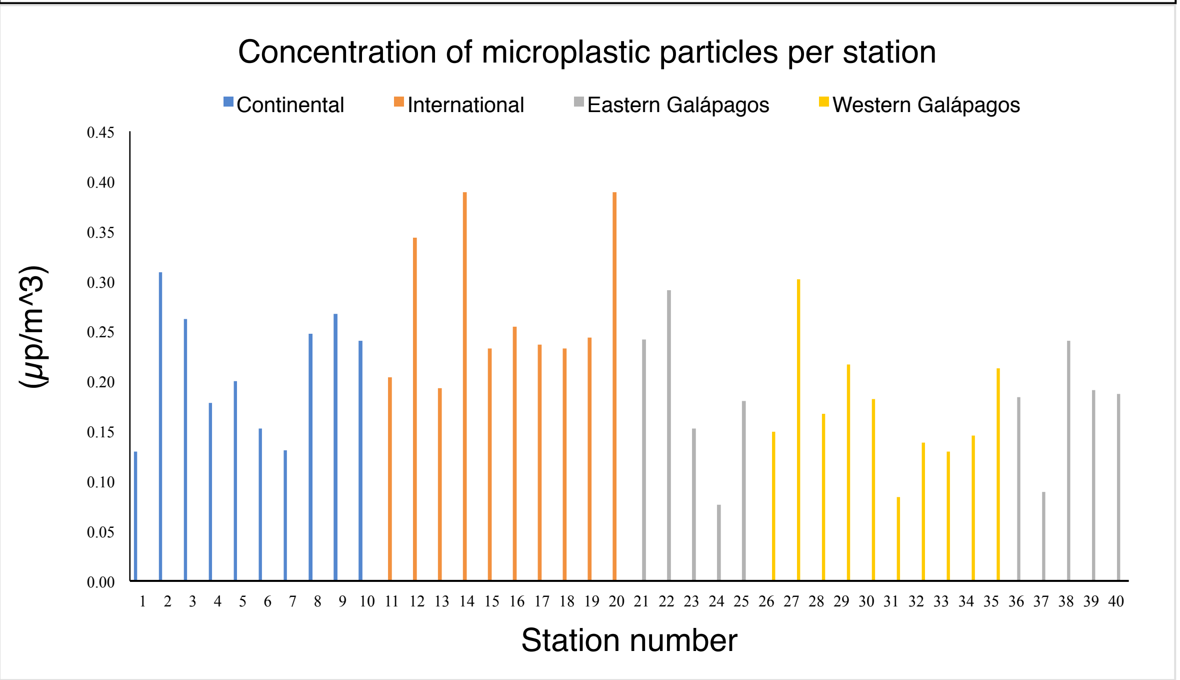

Plankton net 500 µm

One-way ANOVA: Continental waters, International waters, Eastern Galapagos, Western Galápagos

Method

Null hypothesis	All means are equal
Alternative hypothesis	Not all means are equal
Significance level	α = 0.05

Equal variances were assumed for the analysis.

Factor Information

Factor	Levels	Values
Factor	4	Continental waters, International waters, Eastern Galapagos, Western Galápagos

Analysis of Variance

Source	DF	Adj SS	Adj MS	F-Value	P-Value
Factor	3	0,008597	0,002866	5,79	0,002
Error	36	0,017806	0,000495		
Total	39	0,026403			

Model Summary

S	R-sq	R-sq(adj)	R-sq(pred)
0,0222398	32,56%	26,94%	16,74%

Means

Factor	N	Mean	StDev	95% CI
Continental waters	10	0,05018	0,02056	(0,03592. 0,06445)
International waters	10	0,08527	0,02291	(0,07101. 0,09954)
Eastern Galapagos	10	0,05436	0,02437	(0,04010. 0,06863)
Western Galápagos	10	0,05036	0,02091	(0,03610. 0,06463)

Pooled StDev = 0,0222398

Fisher Pairwise Comparisons

Grouping Information Using the Fisher LSD Method and 95% Confidence

Factor	N	Mean	Grouping	
Continental waters	10	0,08527	A	
International waters	10	0,05436		B
Eastern Galapagos	10	0,05036		B
Western Galápagos	10	0,05018		B

Means that do not share a letter are significantly different.

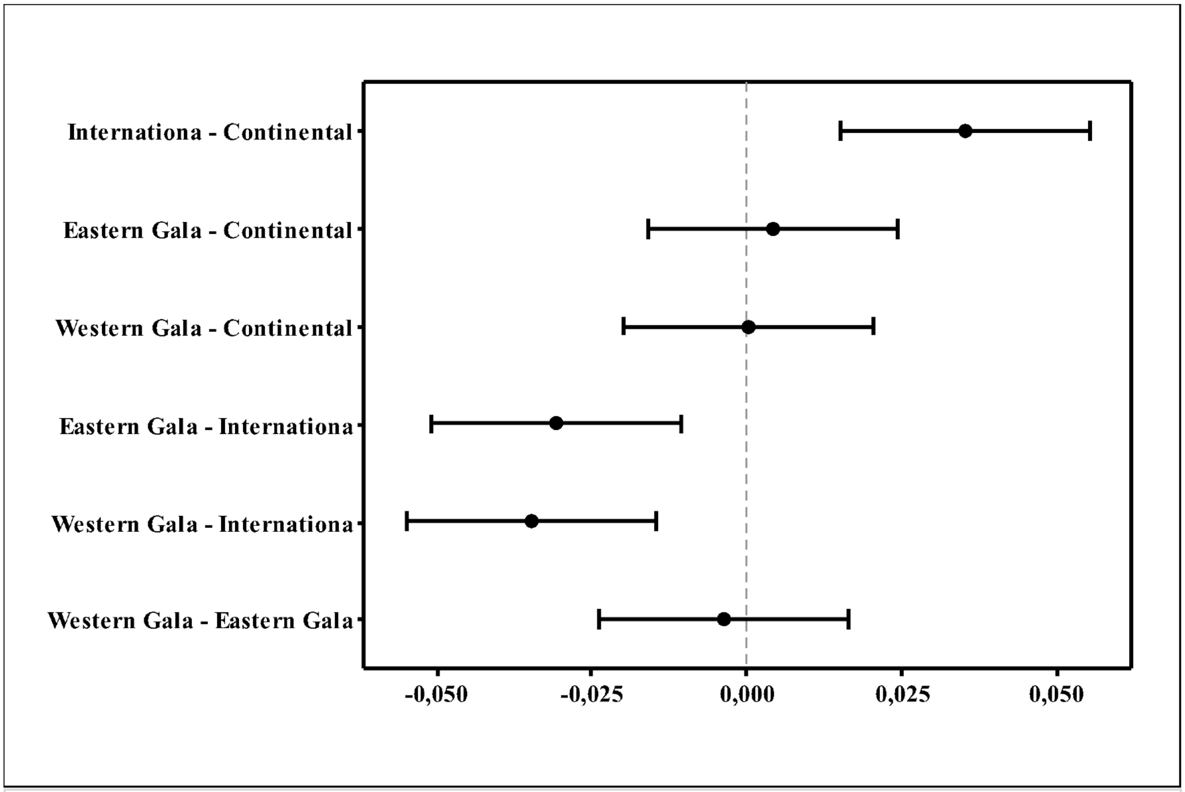

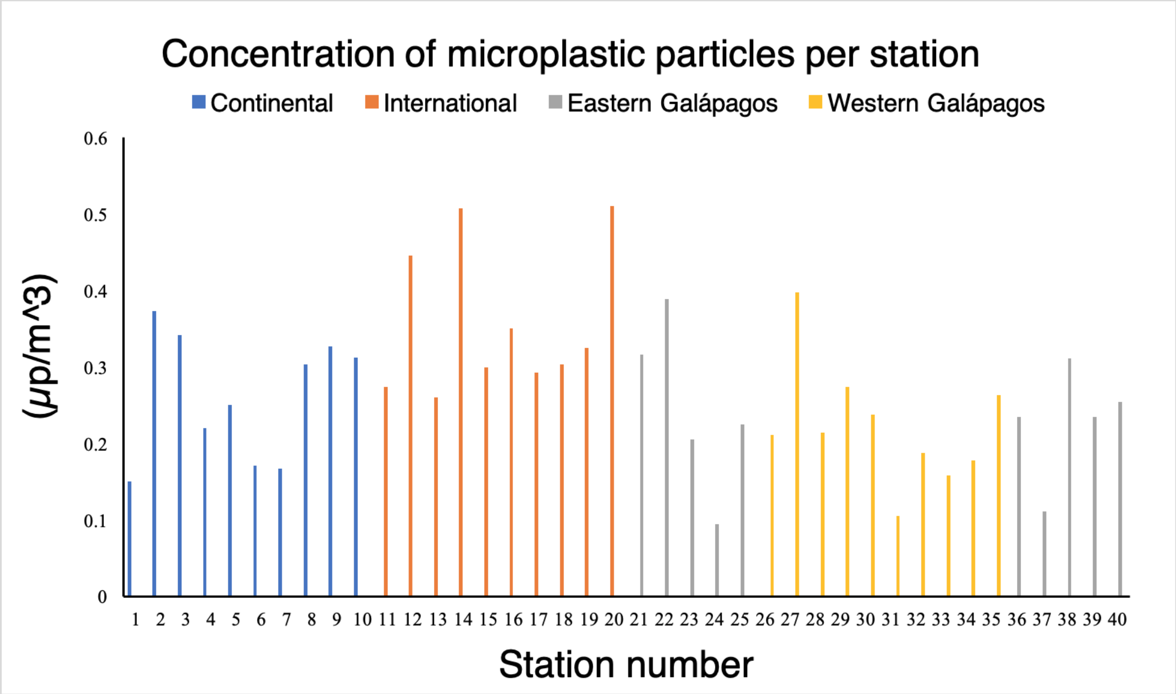

Statistical Analysis of Spatial Interpolations

One-way ANOVA: Continental waters, International waters, Eastern Galapagos, Western Galápagos

Method

Null hypothesis	All means are equal
Alternative hypothesis	Not all means are equal
Significance level	α = 0.05

Equal variances were assumed for the analysis.

Factor Information

Factor	Levels	Values
Factor	4	Continental waters, International waters, Eastern Galapagos, Western Galápagos

Analysis of Variance

Source	DF	Adj SS	Adj MS	F-Value	P-Value
Factor	3	0,1092	0,036389	4,86	0,006
Error	36	0,2695	0,007487		
Total	39	0,3787			

Model Summary

S	R-sq	R-sq(adj)	R-sq(pred)
0,0865282	28,83%	22,90%	12,13%

Means

Factor	N	Mean	StDev	95% CI
Continental waters	10	0,2618	0,0808	(0,2063. 0,3173)
International waters	10	0,3571	0,0952	(0,3016. 0,4126)
Eastern Galapagos	10	0,2376	0,0898	(0,1821. 0,2931)
Western Galápagos	10	0,2229	0,0794	(0,1674. 0,2784)

Pooled StDev = 0,0865282

Fisher Pairwise Comparisons

Grouping Information Using the Fisher LSD Method and 95% Confidence

Factor	N	Mean	Grouping	
Continental waters	10	0,3571	A	
International waters	10	0,2618		B
Eastern Galapagos	10	0,2376		B
Western Galápagos	10	0,2229		B

Means that do not share a letter are significantly different.

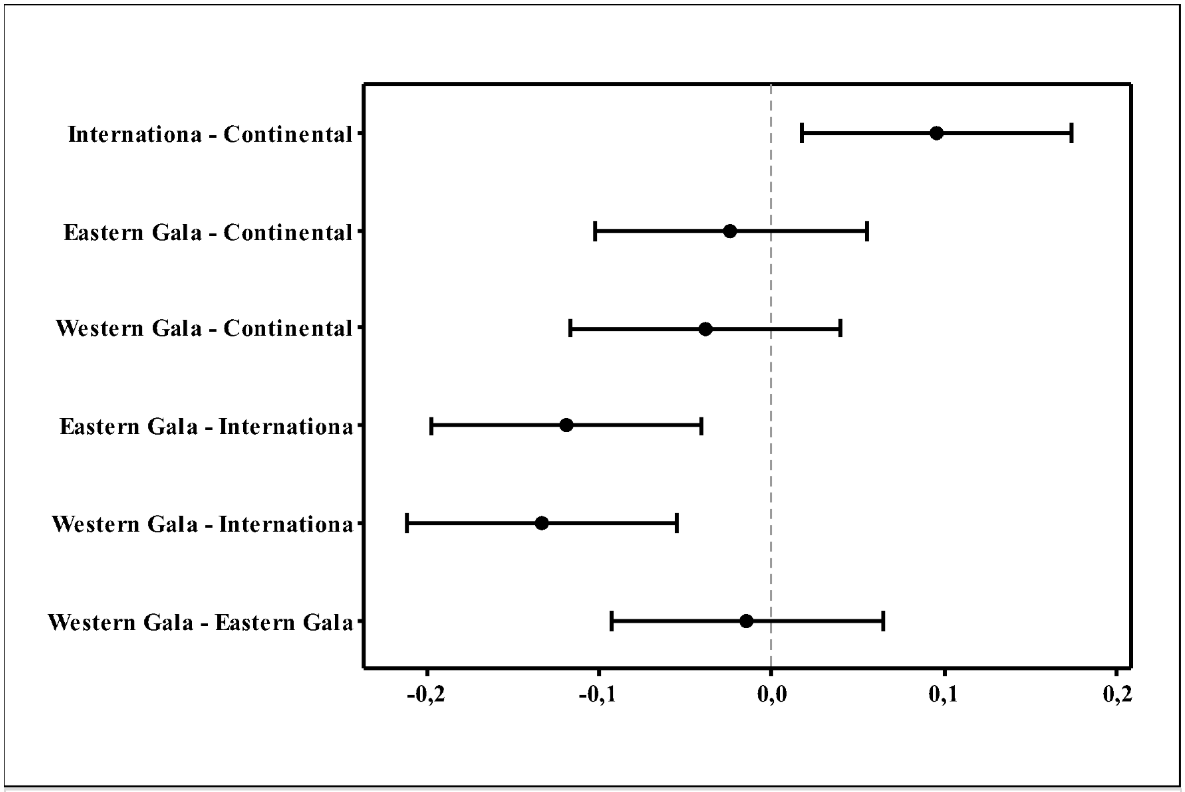
Internationa - Continental
Eastern Gala - Continental
Western Gala - Continental
Eastern Gala - Internationa
Western Gala - Internationa
Western Gala - Eastern Gala
-0,2
-0,1
0,0
0,1
0,2

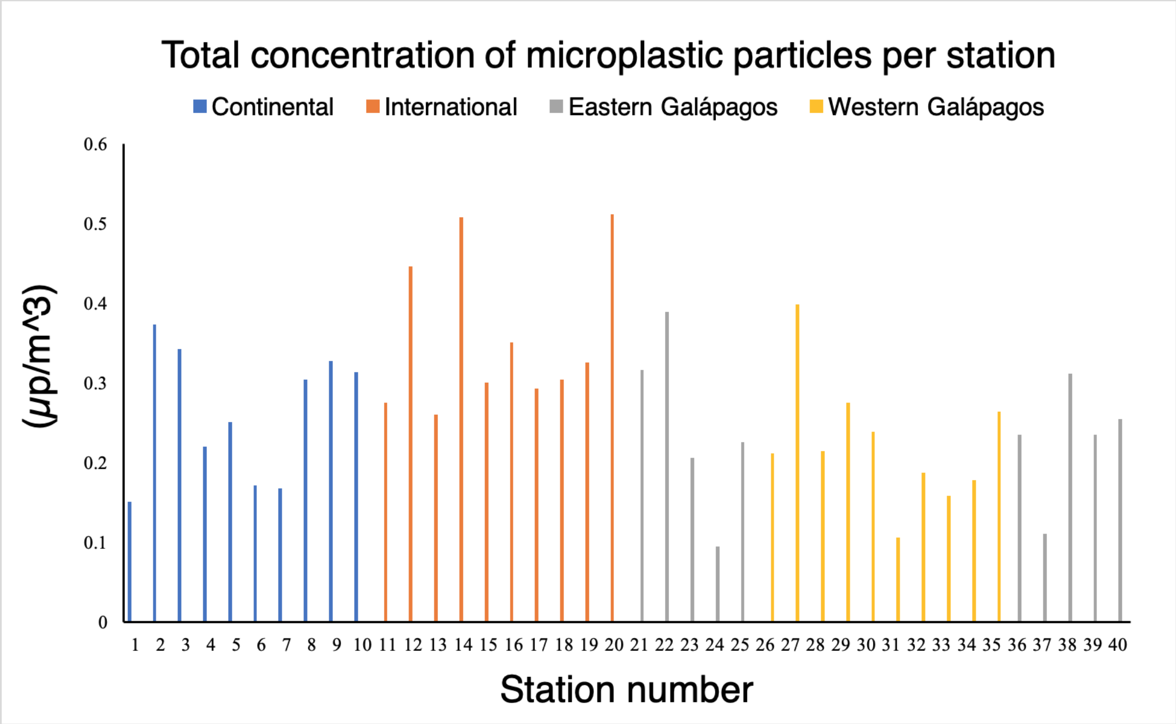
Total concentration of microplastic particles per station
Continental
International
Eastern Galápagos
Western Galápagos
(µp/m^3)
0
0.1
0.2
0.3
0.4
0.5
0.6
1 2 3 4 5 6 7 8 9 10 11 12 13 14 15 16 17 18 19 20 21 22 23 24 25 26 27 28 29 30 31 32 33 34 35 36 37 38 39 40
Station number
